# Cerebral hemodynamics is altered in patients with sleep apnea/hypopnea syndrome

**DOI:** 10.1186/s40064-016-1691-x

**Published:** 2016-01-20

**Authors:** R. Coloma Navarro, P. E. Jiménez Caballero, G. Vega, O. Ayo-Martín, T. Segura Martín

**Affiliations:** Servicio de Neumología, Complejo Hospitalario Universitario, C/Hermanos Falcó, s/n, 02006 Albacete, Spain; Servicio de Neurología, Hospital San Pedro de Alcántara, Cáceres, Spain; Unidad de Cuidados Intensivos, Hospital de la Princesa, Madrid, Spain; Servicio de Neurología, Complejo Hospitalario Universitario, Albacete, Spain

**Keywords:** Cerebral blood flow, Sleep apnea syndrome, Transcranial Doppler, Cerebrovascular reactivity

## Abstract

According to recent epidemiologic studies, patients with sleep apnea/hypopnea syndrome (SAHS) are at increased risk of cardiovascular diseases, including stroke. However, the mechanisms are not well defined. Nocturnal apneas can trigger acute cerebral ischemia in predisposed patients and impaired vasodilatation is present in SAHS, but few studies have explored vascular cerebral dysfunction and often gave inconclusive results. The aims of our study were to assess whether patients with SAHS have impairment of cerebral hemodynamics with respect to controls, and to investigate a possible relationship with clinical data. We studied two groups, one of 76 SAHS patients and another one of 76 non-SAHS subjects matched for age, sex and main cardiovascular risk factors. All participants underwent a daytime transcranial Doppler study of right middle cerebral artery to record cerebral blood flow velocity and cerebrovascular reactivity by means of breath-holding test (BHT). SAHS patients have a reduction in mean cerebral blood flow velocity (MFV) (52 ± 9 vs 60 ± 12 cms/s, p < 0.001) and BHT (31 ± 12 vs 36 ± 11 %, p = 0.005) when compared to non-SAHS controls. Moreover, MFV correlated negatively with the presence of coronary disease, and BHT with female sex and arterial pressure. On the other hand, in the SAHS group, MFV correlated negatively with oxygen desaturation severity. Patients with SAHS have impaired MFV and cerebrovascular reactivity when compared to controls. Interestingly, poorly controlled or unknown hypertension and severe nocturnal hypoxemia caused additional cerebral hemodynamic disturbances to these patients.

## Background

Stroke is a serious and common disorder and a major cause of death worldwide. The pathogenesis of stroke is multifactorial and not fully understood. Although several triggering factors have been already identified, they do no account for all the cases of stroke and new risk factors have been proposed (Díaz and Sempere 2004). Among them, sleep apnea/hypopnea syndrome (SAHS) is now recognized as an important and independent risk factor for cardiovascular disease and stroke, as demonstrated in cross-sectional (Yaggi et al. 2005) and longitudinal (Arzt et al. 2005) studies. Intermittent hypoxia, intrathoracic pressure swings and sympathetic activation, all as a result from recurrent upper airway obstruction, could promote atherogenesis via intermediate mechanisms such as oxidative stress (Lavie 2003), endothelial dysfunction (Ip et al. 2004) or increased procoagulant activity (Chin et al. 1996).

Several specific pathogenic mechanisms could explain the high incidence of stroke in SAHS. Obstructive—but not central—apneas have been shown to cause irregularity in cerebral blood flow velocity (Netzer et al. 1998) and could precipitate nocturnal cerebral ischemia in high-risk patients (Dyken et al. 2004). Moreover, early signs of great-vessel atherosclerosis (Baguet et al. 2005), but also higher incidence of silent brain infarctions (Minoguchi et al. 2007) has been detected in patients with SAHS. These latter results suggest a possible involvement of the cerebral microcirculation in the pathogenesis of stroke in patients with SAHS. These findings are important given that cerebral flow adaptation in response to hemodynamic (autoregulation) or metabolic (cerebral vasoreactivity—CVR) changes occur mainly in the microcirculation, and CVR impairment has been related to an increased risk of stroke (Silvestrini et al. 2000).

Past studies have found a reduction in the vasodilation response, especially in the brachial artery (Ip et al. 2004), in patients with SAHS. However, very few have explored cerebral hemodynamics and vasoreactivity. In addition, these investigations were usually performed in small group of patients and gave conflicting results (Placidi et al. 1998; Diomedi et al. 1998; Urbano et al. 2008). However, in a recent population-based study, Morgan et al. (Morgan et al. 2010) observed a significant positive correlation between the mean level of nocturnal oxygen and cerebrovascular CO_2_ reactivity. Therefore, the objective of this study was to further clarify and define the role of cerebral hemodynamics and cerebral vasoreactivity as potential risk factors for stroke in patients with SAHS. For these investigations we used a large sample of non-selected SAHS patients and compared with a group of non-SAHS controls matched for age and major cardiovascular risk factors.

## Methods

### Study subjects

Patients referred to the Sleep Unit for SAHS with an apnea/hypopnea index (AHI) ≥10/h in overnight full polysomnography were included. Non-SAHS controls were selected from the community and hospital staff; subjects with chronic or heavy snoring, witnessed nocturnal apneas or excessive daytime sleepiness [Epworth Sleepiness Scale (ESS) ≥10] were excluded, although subjects with occasional snoring (less than once a week) were accepted. Both groups were matched for age, sex, smoking status, arterial hypertension (HT), dyslipidemia, diabetes mellitus (DM), coronary disease (CD) and peripheral arteriopathy (PA).

Patients with previous respiratory disease, diurnal hypercapnia (pCO_2_ > 45 mmHg), stroke, arrythmia, cardiac failure or cervical or intracranial arterial stenosis were excluded from the study. Diagnosis of HT, dyslipidemia or DM was done by the primary physician. The Study Protocol was approved by the Hospital General Universitario Ethic Committee. All participants entered the study after giving written informed consent.

### Study measurements

#### Polysomnography

Sleep study was performed with a SleepLab 1000P (Aequitron Medical Inc, Minneapolis, MN, USA) 16-channel polysomnograph. Sleep stages and EEG arousal detection were scored according to standard criteria. Apnea was defined as an airflow cessation of more than 10 s in both the thermistor and nasal cannula. Hypopnea was defined as a 50 % reduction in airflow amplitude of at least 10 s in one airflow signal, with a 4 % oxygen desaturation and/or EEG arousal. AHI was defined as the sum of apneas and hypopneas per hour during the total sleep time. The oxygen desaturation index (ODI) and arousal index (AI) were defined as the respective number of 4 % oxygen desaturations and EEG arousals per hour during the total sleep time. CT_90_ was defined as the percentage of total sleep time with O_2_ saturation under 90 %. All the studies were analyzed by the same observer.

#### Transcranial Doppler study

##### Baseline study

All the subjects were studied in the morning, between 10 a.m. and 12 a.m., in a quiet, semi-dark room, resting in a supine position. SAHS patients were studied within a month of the sleep study. All the tests were performed by the same investigator, who was blind to the results of the sleep study. First, the participants were routinely examined with continuous color-coded duplex ultrasound (Logic Q, GE, linear transducers of 5–8 MHz). If a significant (>50 %) stenosis was found, the patient was excluded from the study. Second, intracranial arteries (middle cerebral arteries—MCA, anterior cerebral arteries—ACA, posterior cerebral arteries—PCA and basilar artery—BA) were identified through the temporal (MCA, ACA and PCA) or suboccipital (BA) windows by transcranial Doppler (TCD) (Multidop B plus DWL, Elektronische Systeme GmbH, Sipplingen, Germany). The peak systolic velocity (PSV) and end diastolic velocity (EDV) were recorded for each artery. The mean blood flow velocity (MFV) was calculated automatically using the formula:

$${\text{MFV}} = ({\text{PSV}} + 2{\text{EDV}})/3.$$

A MFV measurement greater than 95th percentile of our normal laboratory values in any intracranial artery was indicative of cerebral artery stenosis and the subject was excluded from the study.

##### Breath holding test

CVR was assessed by the breath-holding test (BHT). We continuously recorded the right MCA MFV at a depth of 50–55 mm. Participants were asked to hold their breaths for 30 s after a normal inspiration. The CBFV increase was recorded as the percentage increase in post-apnea MFV compared to the baseline value after breath-holding (Ratnatunga and Adiseshiah 1990).

$${\text{BHT}}\,(\% ) = \frac{{{\text{Post-apnea}}\,{\text{MFV}}{-}{\text{Baseline}}\,{\text{MFV}}}}{{{\text{Baseline}}\,{\text{MFV}}}} \times 100$$

Apnea maneuver can induce changes in blood pressure (BP) that could in turn modify cerebral hemodynamics. For that, systolic (SBP) and diastolic blood pressure (DBP) were measured before and immediately after the breath holding, and BP variations were considered in data analysis; mean arterial pressure (MAP) was defined as:

$${\text{MAP}}\, ( {\text{mmHg)}} = {\text{DBP}} + \frac{{{\text{SBP}}{-}{\text{DBP}}}}{3}$$

The difference between post-apnea MAP and baseline MAP was defined as:

$$\Delta {\text{MAP}}\, ( {\text{mmHg)}} = {\text{Post-apnea}}\,{\text{MAP}}{-}{\text{Baseline}}\,{\text{MAP}}$$

### Statistics

The SPSS 22.0 package (SPSS Inc., Chicago, IL) was used for the analysis. All the data were tabulated as mean and standard deviation for quantitative variables and as absolute numbers and percentages in the case of qualitative variables. The Kolmogorov–Smirnov test was used to analyze variables distribution. When necessary, the variables were transformed. In the bivariate analysis, quantitative variables were analyzed using the comparison of means tests (Student’s t or Mann–Whitney’s) and χ^2^ test for categoric variables. The linear association between two quantitative variables was established using the Pearson linear correlation index or Spearman test. Multivariate analysis was performed by multiple linear regression models, including all the independent variables that showed an association with dependent variable with p < 0.2. All other independent variables relevant for the objectives of the study were also included. The inclusion was performed by the “forward” selection method. The strength of the association was measured by the square multiple correlation coefficient adjusted (R^2^). A value of p < 0.05 was considered statistically significant.

## Results

### Main study

Seventy-eight SAHS patients and seventy-six non-SAHS subjects were referred for TCD study. Two SAHS patients were excluded because of significant (>50 %) internal carotid artery stenosis and temporal hyperostosis, respectively. The baseline characteristics of the participants are shown in Table 1. Although subjects were matched for previous cardiovascular risk factors, patients with SAHS had significantly higher values of BMI and both baseline and post-apnea MAP.

**Table 1:** Main characteristics and results of main study

	SAHS group	Control group	*p*
n	76	76	
Gender	67 M/9 F	66 M/10 F	1
Age (years)	48.3 ± 10,5	48.8 ± 11.2	0.8
BMI (kgs/m^2^)	31.2 ± 4.6	26.9 ± 3.9	<0.001
ESS	12.7 ± 4.1	4.1 ± 1.8	<0.001
HT	21 (27.6 %)	18 (23.6 %)	0.5
Current smoking	26 (34.2 %)	27 (35.5 %)	0.8
Dyslipidemia	25 (32.8 %)	21 (27.6 %)	0.4
DM	2 (2.6 %)	2 (2.6 %)	1
CD	4 (5.3 %)	2 (2.6 %)	0.6
Peripheral arteriopathy	0	0	1
Baseline MAP (mmHg)	106 ± 14	98 ± 12	0.001
Post-apnea MAP (mmHg)	109 ± 14	101 ± 11	<0.001
∆MAP (mmHg)	+3.7 ± 10	+2.7 ± 7	0.4
MFV (cms/sg)	52 ± 9	60 ± 12	<0.001
BHT (%)	31 ± 12	36 ± 11	0.005

Results are expressed as mean ± standard deviation or number (percentage). Comparison was made by means of Student´s *t* test for continuous variables and χ^2^ test for categoric variables

*SAHS* sleep apnea/hypopnea syndrome, *M* male, *F* female, *BMI* body mass index, *ESS* Epworth Sleepiness Score, *HT* arterial hypertension, *DM* diabetes mellitus, *CD* coronary disease, *MAP* mean arterial pressure, ∆*MAP* difference between post-apnea MAP and baseline MAP, *MFV* mean flow velocity, *BHT* breath-holding test

On bivariate analysis, the TCD study showed a significant decrease in MFV (52 ± 9 vs 60 ± 12 cms/s, p < 0.001) and BHT (31 ± 12 vs 36 ± 11 %, p = 0.005) in SAHS patients with respect to non-SAHS (Table 1). MFV was also decreased in participants with previous coronary disease and showed a negative correlation with BMI. On the other hand, BHT was lower in women and in dyslipidemic participants, and also showed a negative correlation with age, BMI, and both baseline and post-apnea MAP values (Tables 2, 3).

**Table 2:** Comparison of main cardiovascular risk factors by MFV and BHT

	MFV (cms/sg)			BHT (%)		
	Yes	No	*p*	Yes	No	*p*
Male gender	56 ± 10	58 ± 17	0.560	34 ± 11	28 ± 11	0.025
HT	54 ± 10	57 ± 12	0.213	30 ± 11	34 ± 12	0.057
Current smoking	56 ± 10	56 ± 12	0.814	33 ± 11	33 ± 12	0.963
Dyslipidemia	56 ± 12	56 ± 11	0.996	30 ± 11	35 ± 12	0.017
DM	58 ± 11	56 ± 11	0.633	27 ± 7	34 ± 12	0.300
CD	45 ± 9	56 ± 11	0.022	28 ± 10	34 ± 11	0.285

Results are expressed as mean ± standard deviation

*p* value corresponds to means comparison (Student’s *t*) between each group

*HT* arterial hypertension, *DM* diabetes mellitus, *CD* coronary disease

**Table 3:** Bivariate analysis (Pearson’s linear correlation)

	MFV		BHT	
	r	*p*	r	*p*
Age	−0.097	0.235	−0.162	0.046
BMI	−0.230	0.004	−0.199	0.014
Baseline MAP	−0.073	0.370	−0.286	<0.001
Post-apnea MAP			−0.234	0.004
∆MAP			0.093	0.254

*MFV* mean flow value, *BHT* breath holding test, *BMI* body mass index, *MAP* mean arterial pressure, ∆*MAP* difference between post-apnea MAP and baseline MAP

Multivariate linear regression analysis was performed. The variables included in the model explaining MFV were: case/control status, age, gender, BMI, coronary disease and baseline MAP. The regression coefficient (R^2^) was 0.124. MFV correlated negatively with the presence of SAHS and coronary disease (Table 4). On the other hand, the variables included in the model explaining BHT were: case/control status, age, gender, BMI, dyslipidemia, HT, MFV, baseline and post-apnea MAP, and ∆MAP. The regression coefficient (R^2^) was 0.115. BHT correlated negatively with the presence of SAHS, female sex and baseline MAP (Table 5).

**Table 4:** Multivariate analysis (multiple linear regression) of MFV

MFV	Beta	Confidence interval		*p*
		Low	High	
SAHS group	−7.4	−10.9	−3.8	<0.001
CD	−10.3	−19.4	−1.2	0.026

*MFV* mean flow value, *SAHS* sleep apnea-hypopnea syndrome, *CD* coronary disease

**Table 5:** Multivariate analysis (multiple linear regression) of BHT

BHT	Beta	Confidence interval		*p*
		Low	High	
SAHS group	−4.04	−7.7	−0.3	0.034
Female gender	−5.9	−11.3	−0.44	0.034
Baseline MAP	−0.190	−0.32	−0.05	0.005

*BHT* breath-holding test, *SAHS* sleep apnea-hypopnea syndrome, *MAP* mean arterial pressure

### SAHS group study

The main polysomnographic values of SAHS patients are shown in Table 6. Overall, and although there was no previous selection, this is a group of severe patients with significant impact on nocturnal oximetry.

**Table 6:** Polysomnographic values in SAHS group

	Mean	SD	Range
AHI (number/h)	58.9	24.3	10–122
ODI (number/h)	40.0	28.4	0–92
AI (number/h)	58.4	21.1	19–121
Baseline O_2_ saturation (%)	96.1	1.2	92–98
Mean O_2_ saturation (%)	91.7	2.7	81–96
Minimal O_2_ saturation (%)	74.8	10.1	42–90
CT_90_ (%)	22.2	28.0	0–97

*SAHS* sleep apnea/hypopnea syndrome, *AHI* apnea/hypopnea index, *ODI* 4 % oxygen desaturation index, *AI* EEG arousal index, *CT*_*90*_ total sleep time percentage with O_2_ saturation lower than 90 %

On bivariate analysis (Table 7), MFV correlated negatively with age, ODI and CT_90_, and positively with mean nocturnal O_2_ saturation. BHT correlated negatively with baseline MAP. On the other hand, we found no significative correlation between BHT and main respiratory variables on polysomnography.

**Table 7:** Bivariate analysis (Pearson´s linear correlation) in SAHS group study

	MFV		BHT	
	r	*p*	r	*p*
AHI (number/h)	−0.162	0.162	−0.139	0.232
ODI (number/h)	−0.271	0.018	0.112	0.340
AI (number/h)	−0.085	0.468	−0.096	0.407
Baseline O_2_ saturation (%)	0.198	0.086	0.053	0.649
Mean O_2_ saturation (%)	0.232	0.043	0.048	0.682
Minimal O_2_ saturation (%)	0.169	0.144	−0.128	0.269
CT_90_ (%)	−0.307	0.007	0.061	0.599
Baseline MAP (mmHg)	0.195	0.095	−0.346	0.002
Post-apnea MAP (mmHg)			−0.189	0.103
∆MAP (mmHg)			0.218	0.059

*MFV* mean flow velocity, *BHT* breath-holding test, *AHI* apnea/hypopnea index, *ODI* 4 % oxygen desaturation index, *AI* EEG arousal index, *CT*_*90*_ total sleep time percentage with O_2_ saturation lower than 90 %, *MAP* mean arterial pressure, ∆*MAP* difference between post-apnea MAP and baseline MAP

## Discussion

The vascular risk factors predisposing to stroke in SAHS are not completely defined. In this study, we found that daytime cerebral hemodynamics is abnormal in patients affected by SAHS. Specifically, we observed a significant reduction in MFV and BHT in the SAHS group when compared to controls. These findings are consistent with changes in cerebral circulation, and could indicate a potential greater vulnerability of the cerebral vasculature to stroke injury (Silvestrini et al. 2000).

Reduced MFV in SAHS has been scarcely reported in previous studies and little importance has been given to this finding in most of them (Urbano et al. 2008; Fisher et al. 1992). Lower MFV has been related to cognitive impairment and to the severity of nocturnal hypoxia in SAHS (Findley et al. 1986). However, the cause of this impairment in cerebral blood flow is not known. Cerebral flow velocity may be reduced relative to an increase in peripheral resistance associated with small vessel disease, as it has been suggested in some studies (Minoguchi et al. 2007). On the other hand, we cannot exclude that low MFV could also be produced as a consequence of changes in the cross-sectional area of the middle cerebral artery. Carotid artery dilation, which is present in early stages of atherosclerosis, has been found in SAHS patients free of cardiovascular comorbidities (Drager et al. 2007). Moreover, a recent magnetic resonance imaging study (Coverdale et al. 2014) demonstrates a significant increase in middle cerebral artery diameter following acute hypercapnic challenge in healthy subjects; therefore, it is possible that continuous heavy exposure to nocturnal hypercapnia as occurs in severe SAHS patients could have induced and perhaps maintained such vascular changes. Response to treatment could have helped us to identify pathophysiologic mechanisms leading to this cerebral blood flow impairment. However, there are few available studies. Diomedi et al. (Diomedi et al. 1998) did not report significant changes in MFV after a month of continuous positive airway pressure (CPAP) treatment. We followed part of the present cohort after a mean period of two years of CPAP treatment and what we found was a small, but significant further impairment of MFV (Jiménez-Caballero et al. 2013). In summary, impaired MFV in this cohort of severe SAHS patients could be related to vascular changes, probably involving small vessels and perhaps large intracranial arteries too. According to the few available studies, CPAP treatment did not seem to modify this hemodynamic impairment and this could have prognostic implications since SAHS mainly affects middle-aged adults who may be at increased risk for the rest of their lives. Further studies will be needed to better define the importance of this finding as a cardiovascular risk factor.

We also found CVR impairment in SAHS patients as described in previous studies, most of them including a small number of severe patients. Placidi et al. (Placidi et al. 1998), also using BHT, found a reduction of CVR in a group of patients with severe SAHS when compared to controls. In contrast, four subsequent studies (Urbano et al. 2008; Foster et al. 2009; Furtner et al. 2009; Ryan et al. 2014) did not. However, in a recent population-based study, Morgan et al. (Morgan et al. 2010) describe CVR impairment across a wide spectrum of sleep-breathing disorders in a large sample of 373 participants of the Sleep Heart Health Study cohort; they observed a positive correlation between mean level of arterial oxygen during sleep and vascular cerebral responsiveness to hypercapnia. Many pathophysiological mechanisms can be involved. Endothelial dysfunction and improvement with CPAP have been described in peripheral circulation in SAHS patients (Ip et al. 2004). Moreover, early markers of carotid atherosclerosis have been found and a positive response to treatment has been reported (Drager et al. 2007); therefore, endothelial dysfunction described in peripheral and extracranial arteries could also involve intracranial arteries. On the other hand, high sympathetic activity by itself could cause CVR impairment, as it has been proved in healthy subjects (ter Laan et al. 2013). High nocturnal sympathetic activity is almost always present in severe SAHS patients and may remain in the morning, and previous studies show that CPAP treatment can normalize morning levels of catecholamines after a single night of treatment (Minemura et al. 1998). Interestingly, Diomedi et al. (Diomedi et al. 1998) also reported an early improvement in CVR in patients after a first night with CPAP, and this effect was maintained a month later. We also found a significant improvement of CVR in our SAHS cohort after two years of follow-up (Jiménez-Caballero et al. 2013), together with significantly lower diastolic blood pressure levels. Therefore, although endothelial dysfunction is present in severe SAHS patients and could probably contribute to vasodilatation impairment, the early improvement of CVR with CPAP described in interventional studies suggests that high sympathetic activity could also account for a big part of the hemodynamic disturbance.

In the present study, we also found that decrease in CVR was independently related to high baseline BP values, indicating either unknown or poorly controlled hypertension. Chronic hypertension causes endothelial dysfunction and vasodilatation impairment. Moreover, one recent well-designed study (Drager et al. 2009) has found a significant increase in early markers of carotid atherosclerosis in hypertensive SAHS patients compared to healthy controls, non-hypertensive SAHS or hypertensive non-SAHS subjects respectively, suggesting that SAHS and hypertension could have an accumulative effect in vascular damage in these patients.

Another important finding from our study is the association of impaired CVR with female sex. Even though female sex is not a risk factor per se, greater reduction in CVR has been found in post-menopausal women as compared to premenopausal women and men of all ages (Matteis et al. 1998). Moreover, impaired endothelial function associated with sleep-breathing disorders has also been described in women, but not in men, in a cohort study of 193 participants (Faulx et al. 2004). In our study, women were significantly older than men (mean age 54 ± 8 vs 47 ± 10 years, p = 0.006, data not shown) and most of them would probably be menopausal, although these data were not recorded in our questionnaire.

In our SAHS group, MFV reduction was related to oxygen desaturation severity, but not to AHI. Oxygen desaturation severity has also been correlated with the presence of early markers of carotid atherosclerosis (Baguet et al. 2005) and of silent brain infarction (Minoguchi et al. 2007) in SAHS patients. In the study by Morgan et al. (Morgan et al. 2010), nocturnal oxygen saturation was also a more important predictor of cerebral vascular dysfunction than was AHI. We did not find any correlation between BHT and respiratory variables in our SAHS patients; as most of them had severe disease, we believe that this could explain the absence of significant correlation with hemodynamic impairment; in this sense, it would be interesting to perform this study in a sample of less severe SAHS patients.

Our study has some potential limitations. First of all, a limitation in the TCD methodology is the correlation of the CBFV with cerebral flow when the MCA diameter remains constant at the point of insonation. Although previous studies did not find any significant difference in the diameter of the artery during changes in end-tidal CO_2_, a more recent paper (Coverdale et al. 2014) describes a significant increase in cross-sectional area of MCA during hypercapnia in healthy volunteers. Second, we did not perform a sleep study in the non-SAHS group; therefore, we cannot rule out an AHI > 5/h in some cases. However, since none of these subjects reported habitual snoring, witnessed apneas or excessive daytime somnolence, this possibility is less likely. This is supported by previous studies demonstrating that only 6 % of SAHS patients are not snorers (Viner et al. 1991). Third, and despite a random selection process, the mean baseline and post-apnea BP values were significantly higher in the SAHS group, suggesting the possibility of undiagnosed HT in some cases. Chronic HT could have explained the low values in MFV and BHT. However, the differences between groups remained significant after adjusting for these confounding factors.

In summary, our study highlights the impairment of daytime cerebral hemodynamics in patients with severe SAHS. These findings point out to possible functional and structural changes in cerebral microcirculation and perhaps in large intracranial arteries too as a potential mechanism to help to explain the increased risk of stroke in these patients. Further studies will be needed to identify risk groups more accurately and to evaluate possible changes in cerebral hemodynamics after therapeutic intervention.
